# Efficient Privacy-Preserving Face Recognition Based on Feature Encoding and Symmetric Homomorphic Encryption

**DOI:** 10.3390/e28010005

**Published:** 2025-12-19

**Authors:** Limengnan Zhou, Qinshi Li, Hui Zhu, Yanxia Zhou, Hanzhou Wu

**Affiliations:** 1 School of Electronic and Information Engineering, University of Electronic Science and Technology of China, Zhongshan Institute, Zhongshan 528402, China; lmnzhou@zsc.edu.cn; 2 School of Computer and Software Engineering, Xihua University, Chengdu 610039, China; 3 School of Cyberspace Security (School of Cryptology), Hainan University, Haikou 570228, China; zhuhuifree@hainanu.edu.cn; 4 School of Information Science and Technology, Xizang University, Lhasa 850000, China; 5 School of Communication and Information Engineering, Shanghai University, Shanghai 200444, China; hanzhou@shu.edu.cn

**Keywords:** face recognition, privacy, homomorphic encryption

## Abstract

In the context of privacy-preserving face recognition systems, entropy plays a crucial role in determining the efficiency and security of computational processes. However, existing schemes often encounter challenges such as inefficiency and high entropy in their computational models. To address these issues, we propose a privacy-preserving face recognition method based on the Face Feature Coding Method (FFCM) and symmetric homomorphic encryption, which reduces computational entropy while enhancing system efficiency and ensuring facial privacy protection. Specifically, to accelerate the matching speed during the authentication phase, we construct an N-ary feature tree using a neural network-based FFCM, significantly improving ciphertext search efficiency. Additionally, during authentication, the server computes the cosine similarity of the matched facial features in ciphertext form using lightweight symmetric homomorphic encryption, minimizing entropy in the computation process and reducing overall system complexity. Security analysis indicates that critical template information remains secure and resilient against both passive and active attacks. Experimental results demonstrate that the facial authentication efficiency with FFCM classification is 4% to 6% higher than recent state-of-the-art solutions. This method provides an efficient, secure, and entropy-aware approach for privacy-preserving face recognition, offering substantial improvements in large-scale applications.

## 1. Introduction

As a strategic technology shaping the future, facial recognition has become an integral part of modern society, with its widespread adoption leading to an increase in the entropy of personal data management. The technology exerts a profound influence on economic development, social progress, and daily life. Facial recognition is extensively used in security monitoring, identity verification, social networks, mobile payments, and various other domains [1]. It offers significant convenience in daily life, demonstrates substantial business value, and holds immense development potential. Numerous countries have placed considerable strategic emphasis on facial recognition technology, leading to the emergence of many scientific research institutions, vigorous efforts by leading technology companies, and the rapid rise of new enterprises in the field. However, facial recognition technology has also sparked significant controversy regarding privacy protection, as it enables the creation of searchable biometric databases by capturing and storing facial biometrics. The entropy of such systems increases significantly when biometric data is mishandled or improperly protected, heightening the risk of data misuse or leakage, which can pose a severe threat to personal privacy [2]. Therefore, privacy protection technologies are crucial for reducing the entropy of personal information security, ensuring that individuals’ sensitive data remains safe from misuse and unauthorized access.

Recent privacy-preserving facial recognition schemes primarily protect image privacy through the methods of image obfuscation, adding adversarial noise, and identity transformation. Image obfuscation [3,4] introduces specific noise or alterations to the image, making it difficult for the system to extract meaningful features from the obfuscated image. However, this method suffers from significant information loss, weak resistance to adversarial attacks, and lower accuracy [5]. Adding adversarial noise [6,7] involves introducing small perturbations to the input, such as an image, causing the model to make significant errors in prediction or classification, thereby defending against attackers. However, this may lead to a decline in classifier performance and impact recognition accuracy [8]. Identity transformation [9,10] uses techniques to alter an individual’s facial features, making their facial image appear as that of another person. However, the transformation process is complex and requires high computational resources [11]. The limitations of the aforementioned three methods can be effectively addressed by homomorphic encryption algorithms [12]. Therefore, this paper focuses on utilizing homomorphic encryption to achieve facial privacy protection.

In recent years, several studies have utilized homomorphic encryption techniques to achieve facial privacy protection [13,14]. These papers combine homomorphic encryption with other algorithms to ensure user facial privacy, optimize recognition performance, and demonstrate the security of their proposed solutions. Unfortunately, these methods all face the issue of performing full-text search over ciphertext on the server side. While using identity IDs can resolve this problem, the issue of how to store identity IDs is not suitable for practical systems [15]. Therefore, the focus of this study is on achieving fast matching rates and high recognition accuracy while protecting user facial privacy. This requires overcoming three key challenges:

- What facial information should be encrypted? Suppose a color facial image is 1024 × 1024 × 3 bytes. If homomorphic encryption is directly applied to the image, it would require encrypting 1024 × 1024 × 3 = 3,145,728 times. However, if the facial feature vector (1024 bytes after dimensionality reduction) is encrypted, it would only require 1024 encryptions.
- Which encryption algorithm should be chosen to encrypt facial feature information? When the server performs facial matching similarity calculations or processes result bits, using plaintext for these operations risks data leakage, with the final decision-making power residing on the server. What encryption algorithm can ensure the privacy of the user’s facial data while transferring the decision-making authority for face verification to the client? If a homomorphic encryption algorithm is adopted, which includes partial homomorphic encryption, fully homomorphic encryption, and symmetric homomorphic encryption, which homomorphic encryption scheme should be selected to encrypt facial feature vectors in this approach?
- How can facial feature templates be searched in ciphertext? The server performs similarity computation on ciphertext, requiring the corresponding facial feature ciphertext to be retrieved first. Using a brute-force search approach would reduce the efficiency of facial matching. Therefore, a retrieval method is needed to improve matching efficiency while preventing facial privacy leakage.

In this paper, to tackle the three challenges outlined above, we introduce FFCM and integrate it with homomorphic encryption algorithms to develop a privacy-preserving, lightweight facial recognition scheme. The main contributions of this paper are as follows:

- This paper encrypts the user’s facial features instead of the facial image itself, which significantly reduces the encryption time and enhances the matching efficiency. Principal Component Analysis (PCA) is used to reduce the dimensionality of facial images, which not only decreases storage requirements but also shortens the encryption time.
- A lightweight symmetric homomorphic encryption algorithm is employed to encrypt facial features and decrypt similar ciphertexts. The encryption and decryption operations of the symmetric homomorphic encryption algorithm are carried out on the client side. Homomorphic encryption supports modular addition and multiplication on ciphertexts within a finite field, making it well-suited for the cosine similarity algorithm. This approach ensures that the server can compute the similarity of facial features without accessing the user’s private facial data.
- A facial feature encoding method (FFCM) is proposed, which constructs a facial classification model and assigns corresponding encodings at each level of an N-ary tree. Through this N-ary tree, each leaf node stores a set of multiple facial feature vectors, with each user’s face being assigned a corresponding feature encoding. During the authentication phase, the server can quickly search within the classification set using the facial feature encoding (rather than performing a full-text search), thereby significantly accelerating the server-side matching process. Experimental results show that the face authentication efficiency with FFCM classification is 4.65% higher than that of a similar solution using homomorphic encryption [12], and 6.11% higher than [16].

In summary, to address the three challenges, this paper proposes a privacy-preserving facial recognition scheme that combines the facial feature coding method (FFCM) and symmetric homomorphic encryption technology. Specifically, for the first challenge, Principal Component Analysis (PCA) is used to convert facial images into compact feature vectors to minimize the encryption overhead. For the second challenge, a lightweight symmetric homomorphic encryption scheme is adopted to perform similarity calculations in the encrypted domain while maintaining data confidentiality. For the third challenge, the proposed FFCM constructs an N-ary feature tree to effectively narrow the search space, thereby significantly accelerating the ciphertext matching speed without compromising privacy.

## 2. Related Work

Existing privacy-preserving facial recognition methods can be categorized into three approaches: anonymization methods, encryption methods, and differential privacy methods.

Traditional methods for facial anonymization typically involve techniques such as blurring, pixelation, and noise addition. However, these processes often result in the loss of semantic information, which can reduce detection and recognition accuracy. As a result, several learnable anonymization methods [9,17,18,19,20,21] based on face swapping have been proposed to retain essential information for various tasks. However, the generated faces may overlap with real faces. For example, ref. [22] proposed an anonymized dataset for Re-ID, where facial regions are detected and blurred. However, concerns about privacy leakage in non-facial areas remain, limiting the system’s functionality. Ref. [23] introduced a joint learning reversible anonymization framework that can reversibly generate anonymized full-body images for pedestrian re-identification (Re-ID) tasks. However, in the ISED architecture, each identity requires the allocation of a specific key, which may face key management challenges in large-scale systems.

Privacy protection based on encryption involves using mathematical algorithms to encode data, ensuring that unauthorized users cannot access the original values. Erkin et al. [24] demonstrated that privacy-preserving facial recognition can be achieved by employing secure multi-party computations and designing an efficient protocol. This protocol enables the matching of encrypted facial images with a facial template database and provides guidelines for parameter size selection to achieve effective classification rates. However, due to the limitations of online communication and the high computational cost associated with cryptographic processes, the protocol is challenging to implement in practical large-scale applications. Sadeghi [25] proposed a method that combines homomorphic encryption with garbled circuits, leveraging established cipher building blocks. This approach significantly enhances the communication and computational efficiency of Erkin et al.’s [24] method, making it more suitable for handling large datasets. However, encrypting facial images for recognition purposes increases computational costs for both users and servers. Xiang et al. [26] were the first to propose a privacy-preserving facial recognition protocol that effectively safeguards personal privacy by employing encryption algorithms and outsourcing the computation. The protocol significantly improves upon previous efforts by reducing online computing costs, thanks to the outsourcing of substantial computational tasks to cloud servers with greater processing power. Subsequently, Wang and Nakachi [27] proposed a low-complexity encryption algorithm, addressing the high computational complexity typically introduced by ciphertext. They also established a distributed learning framework to address facial recognition challenges under privacy protection. Liu et al. [28] match encrypted face queries with clustered faces in the repository, significantly reducing computational complexity.

The core idea of differential privacy (DP) is to introduce controllable noise (e.g., Laplacian or Gaussian noise) during data processing stages—such as facial feature extraction or model training—to achieve data randomization. Its key mechanism lies in using a “privacy budget (ϵ)” to control the noise intensity: a smaller ϵ results in stronger noise, offering a higher degree of privacy protection but potentially reducing data utility (e.g., feature discriminability); conversely, a larger ϵ leads to weaker noise, improving data utility at the cost of lower privacy protection. This inherent tension is known as the “privacy–accuracy trade-off” [29].

In the field of face recognition, existing research has explored ways to mitigate this trade-off. For instance, Mao et al. [30] integrated a DP mechanism into a deep convolutional neural network-based face recognition model by adding Gaussian noise during gradient updates in training, achieving acceptable recognition performance while preserving data privacy. Li et al. [31] designed a lightweight DP face recognition algorithm that optimizes the noise injection location—introducing noise only at the feature output layer. This approach enhances both the comprehensiveness of privacy protection and the usability of facial images by minimizing the impact of noise on recognition accuracy. Inspired by the ‘lack of correlation in the median of image matrices’, Liu et al. [32] proposed a Sliding Window Release (SWR) algorithm. Compared to the conventional method of directly adding Laplacian noise to images, the SWR algorithm applies noise smoothing within local windows, effectively reducing the distortion of sensitive facial features (e.g., contours of facial organs) and better preserving discriminative facial information. However, existing DP-based approaches still share common limitations: although the studies mentioned above have alleviated the privacy–accuracy trade-off through optimized noise strategies, the fundamental nature of DP makes the introduction of noise unavoidable. When dealing with high-resolution facial images or scenarios with complex pose variations, even optimized noise injection may interfere with subtle facial features (such as eye corner texture or nose bridge curvature), potentially leading to an increase in the False Rejection Rate (FRR). Moreover, for applications requiring real-time responses (e.g., mobile identity verification), the computational overhead of noise injection in some DP schemes may still cause delays, making it difficult to meet efficiency requirements. To provide a systematic comparison of the existing techniques discussed above, Table 1 summarizes the key features, advantages, and limitations of the three main categories: anonymization methods [9,17,18,19,20,21,22,23], encryption methods [24,25,26,27,28], and differential privacy methods [29,30,31,32].

Recently, Sun and Liu [33] conducted a comprehensive review of privacy-preserving facial recognition, covering protection strategies for data generation, inference, and template storage. Their research indicates that cryptographic-based methods, especially those relying on homomorphic encryption, can provide strict privacy guarantees, but often have high computational costs, which limit their practical application in large-scale systems. In this context, our work is particularly dedicated to enhancing the efficiency of encrypted inference by combining lightweight symmetric homomorphic encryption with structured indexing based on FFCM, in order to reduce the cost of ciphertext matching while maintaining strong privacy protection.

## 3. Preliminaries

This section will introduce the preliminaries of eigenfaces, symmetric homomorphic encryption, and FFCM, the system model and the security requirements of our scheme. All the notations mentioned in our proposed scheme are defined in Table 2.

### 3.1. Eigenface

Eigenface [34] is a facial feature extraction method based on Principal Component Analysis (PCA), widely used in face recognition and classification tasks. It employs eigenvalue decomposition techniques from linear algebra to project high-dimensional facial image data into a lower-dimensional feature space, preserving the most critical features with minimal information loss. PCA is a commonly used data analysis technique. It transforms the original data into a set of linearly independent representations through linear transformations, which can be used to extract the main components of the data. Suppose $\Phi =\left\{\Phi_{1},\Phi_{2},\ldots ,\Phi_{N}\right\}$ is a grayscale face image dataset consisting of *N* facial images, each of size m∗n. To apply PCA, we first flatten each image into a one-dimensional feature vector $x_{i}$ of size m∗n, and then stack all the feature vectors to form a data matrix $K\in R^{(m\cdot n)*N}$, where each column represents a flattened image. We then subtract the mean vector μ of all images from each image’s feature vector. The mean vector μ is computed by averaging the pixel values across all images. After centering, we obtain a centered data matrix $K^{\prime }$, where the mean of each feature dimension is zero. Then, the covariance matrix $C=\frac{1}{N-1}K^{\prime }K^{\prime T}$, where $K^{\prime }$ is the centered data matrix. Subsequently, we compute the eigenvalues $\mu_{i}$ and eigenvectors $\eta_{i}$ of the covariance matrix C. The eigenvectors are sorted by their corresponding eigenvalues in descending order, and the top k eigenvectors corresponding to the largest eigenvalues are selected. These top *k* eigenvectors are then stacked into a new matrix $W_{k}\in R^{(m\cdot n)*k}$, which represents the reduced feature space after PCA. By multiplying the centered data matrix $K^{\prime }$ with the projection matrix $W_{k}$, we obtain the reduced-dimensional feature matrix $K_{PCA}=K^{\prime }W_{k}$, with dimensions k×N, representing the PCA-reduced facial features.

Although feature extractors based on deep learning usually achieve higher recognition accuracy, their high-dimensional representation forms and complex parameter structures are not suitable for the homomorphic encryption environment. Therefore, the feature face method based on Principal Component Analysis is adopted to achieve an effective balance among recognition performance, computational efficiency, and encryption feasibility. Although Principal Component Analysis (PCA) was used in this study due to its low-dimensional characteristic that is convenient for encryption, the proposed framework is not limited to PCA. Instead, it can be extended to feature encoders such as ArcFace or FaceNet, which are based on modern deep learning. This integration is feasible, but it will significantly increase the size of the ciphertext and the computational cost. Therefore, it is left as a future optimization direction.

### 3.2. Symmetric Homomorphic Encryption

In this study, we employed a lightweight symmetric homomorphic encryption (SHE) scheme based on integer factorization cryptography, which supports addition and multiplication operations on encrypted integers. The key size of this scheme is 2048 bits, and its security level is comparable to a 128-bit symmetric security level. Moreover, under the assumption that integer factorization remains difficult to compute, it is sufficient to resist known practical attacks.

The security of the adopted SHE scheme depends on its semantic security feature, which ensures that the ciphertext does not reveal any information about the corresponding plaintext. Based on this assumption, even if the attacker observes multiple ciphertexts or performs selected plaintext queries, the encrypted facial feature vectors and similarity values cannot be distinguished from random values.

As a symmetric homomorphic encryption algorithm, it mainly consists of three algorithms: key generation, encryption and decryption. Below, we describe them in detail [35,36].

(1) Key Generation: The key generation algorithm KeyGen() takes a security parameter as input and generates two large prime numbers *j* and *k*, where *j* is significantly larger than *k*. Then, a random integer *r* is selected from $Z_{j}^{*}$, namely *r*←$Z_{j}^{*}$. The secret key is generated as SK=(r,k).

(2) Encryption: Given a message *m*, firstly select a random number *p* that satisfies p+k<j. Then, we use the secret key SK=(r,k) and a parameter α generate the ciphertext of *m*:

(1) $$c=E\left(SK,m,\alpha \right)=r^{\alpha }\left(pk+m\right)\;mod\;j$$

Parameter α is a randomly selected confidentiality factor that is used in each encryption operation to introduce probabilistic behavior and enhance the indistinguishability of the ciphertext. This randomness ensures semantic security by preventing the same plaintext from generating the same ciphertext.

(3) Decryption: A ciphertext c=Enc(m) can be decrypted with the secret key SK=(r,k) as

(2) $$m=(cr^{-\alpha }\;mod\;j)\;mod\;k$$

*(verify)*:

(3) $$\begin{array}{cc} cr^{-\alpha }\;mod\;j & =\left(r^{\alpha }\left(pk+m\right)\;mod\;j\right)r^{-\alpha }\;mod\;j\\ \; & =\;(pk+m)\;mod\;j \end{array}$$

Since j≫k, it follows that pk+m<j. Therefore, $(cr^{\alpha }\;mod\;p)=pk+m$ holds over Z. Consequently, the message *m* can be recovered.

The security of the proposed symmetric homomorphic encryption scheme is based on the computational difficulty of the integer factorization problem. Specifically, compromising the confidentiality of the ciphertext or recovering the plaintext without the key can both be reduced to the problem of integer factorization for large composite numbers. For attackers with polynomial-time computing capabilities, this problem is generally considered unsolvable. Therefore, under the established assumptions, this scheme provides strong cryptographic security and meets the semantic security requirements against chosen plaintext attacks.

In order to facilitate the reproduction of the experimental results and to better understand the application method of homomorphic encryption in the proposed scheme, the organization of the encryption computing process is as follows: Firstly, the client preprocesses the extracted facial feature vectors and converts them into a fixed-length representation based on integers that is suitable for encryption. Secondly, the symmetric homomorphic encryption scheme is used to encrypt the preprocessed feature vectors and transmit them to the server. Thirdly, the server directly performs similarity calculations on the encrypted queries and the encrypted templates stored in the ciphertext domain without accessing any plaintext information. Finally, the encrypted similarity results are returned to the client for decryption and final identity verification.

### 3.3. FFCM (Face Feature Coding Method)

To address the issues of low efficiency and slow processing time in server-based face matching, we propose a novel Face Feature Coding Method (FFCM) based on face feature classification. The core concept of this approach is to integrate a classification technique with an N-ary tree structure. Each leaf node of the N-ary tree corresponds to a classification subset, which remains empty during the initialization phase. Each layer of the N-ary tree corresponds to a distinct classification model. During the registration stage, all facial features are passed through the first-layer classification model at the root node. Ultimately, each user’s facial features are assigned to the appropriate leaf node, with each leaf node being associated with a unique code. Next, we describe our FFCM with an example.

**Figure 1:**
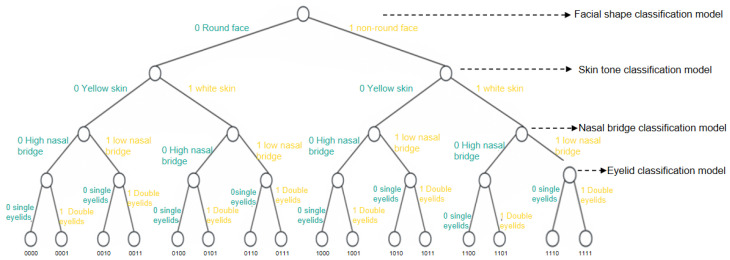
Binary tree for face feature classification.

Figure 1 shows a binary tree with a depth of 5 (N = 2). The root node utilizes a face shape classification model, dividing the facial features into two categories: round face and non-round face according to the face shape. The child node corresponding to the round face category is assigned the number 0, while the child node for the non-round face category is assigned the number 1. The sub-nodes for round faces and non-round faces are further processed using a skin color classification model. This model categorizes the facial features into yellow skin and white skin categories based on skin color. The sub-node corresponding to yellow skin is assigned the number 0, while the sub-node for white skin is assigned the number 1. Using the nose bridge classification model, facial features are categorized into two groups: high nose bridge and low nose bridge. The child node for the high nose bridge category is assigned the number 0, while the child node for the low nose bridge category is assigned the number 1. Based on the eyelid classification, facial features are categorized into two groups: single eyelid and double eyelid. The child node for single eyelid is assigned the number 0, while the child node for double eyelid is assigned the number 1. Finally, the child nodes corresponding to single eyelid and double eyelid become the leaf nodes of the binary tree, and no further face classification models are applied to these nodes.

Thus, after applying the four layers of face classification models, all facial features are categorized into 16 distinct types, each stored in the corresponding leaf nodes. For example, the leaf node number 1001 represents the face features for “non-round face, yellow skin, high nose bridge, double eyelids.” It should be noted that while this article uses a binary tree for illustrative purposes, the actual implementation is not restricted to a binary structure and can accommodate trees with varying values of *N*.

### 3.4. System Model

**Figure 2:**
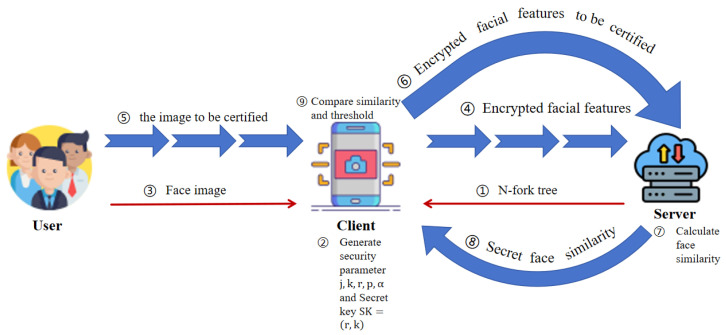
System framework.

Our system model includes three entities—user, client, and server—as shown in Figure 2.

User: The user is an untrusted entity. During the registration and authentication phase, users upload their facial images to the client through sensor devices.

Client: The client is a trusted entity. The function of the client is to generate security parameters; reduce the dimensionality of facial images uploaded by users, extract features for classification, and encrypt them before uploading them to the server; and compare facial similarity against the threshold.

Server: The server is an untrusted entity. The server constructs an N-tree for classifying facial features and sends it to the client. It is responsible for storing the encrypted facial features uploaded by the client during the registration phase; it calculates the similarity in the ciphertext state during the authentication phase.

The basic idea behind this paper is to upload face feature encryption to the server for similarity matching, and then send back to the client to decrypt the similarity, and the client will judge whether it passes. In this paper, firstly, the server uses several classification models based on the neural network model created using the Keras library to construct a new face feature classification method, and fuses the classification method with an N-ary tree to build a Face Feature Coding Method (FFCM). Each leaf node of the N-tree corresponds to a classification subset, which is empty during the initialization phase. Finally, the server publishes the FFCM to the client. Second, the user uploads the face image to the client through the sensing device. After the client obtains the face image, the face feature is extracted by VGG16, the dimension is reduced, and the face feature is encoded by FFCM. The VGG16 network is only used to extract the rough facial features required by the FFCM classifier. Then, the client uses the symmetric homomorphic encryption algorithm to encrypt the face feature value and finally sends the ciphertext and code of the face feature to the server. The server finds the corresponding classification subset according to the encoding and stores the ciphertext of the face feature into the set. We then set key generation parameters on the client. In the authentication phase, as in the registration phase, the client receives the eigenvalue encoding and ciphertext of the user’s face image and sends them to the server. According to the code, the server quickly matches the subset where the face resides, performs ciphertext calculation on the ciphertext awaiting authentication and ciphertext in the subset one by one, obtains the ciphertext set of similarity, and returns the set to the client. After decryption, the client obtains the similarity between the face feature and the elements in the classification set, and determines whether there are elements in the similarity set that are less than the threshold value. If yes, the authentication succeeds and the agreement is terminated. Otherwise, the authentication result is sent to the server. After receiving the request, the server repeats the preceding method in other subsets according to the principle of proximity. If no element smaller than the threshold is found in all subsets, authentication fails.

### 3.5. Threat Model and Trust Assumptions

In the proposed system, users, clients and servers assume different roles and have clear trust assumptions. Users are solely responsible for providing the original facial images and do not participate in any encryption operations. The client is regarded as completely trustworthy. Before transmitting the data to the server, it performs facial feature extraction, FFCM encoding, and encryption processing. The server is assumed to be honest but curious, meaning it will correctly execute all protocol operations, but may attempt to infer sensitive information from the stored encrypted templates, transmitted messages, or classification codes.

The communication channel between the client and the server is assumed to be insecure and may suffer from eavesdropping, replay, and spoofing attacks. However, attackers cannot crack standard encryption algorithms or solve the underlying complex mathematical problems in polynomial time.

### 3.6. Security Requirements

Authentication scheme should satisfy the following security requirements.

- *Mutual authentication*: Two-way authentication between the client and the server is achieved through a challenge–response interaction mechanism based on dynamically generated session identifiers. In each authentication session, the client initiates communication by sending an authentication request containing a freshly generated session identifier. After receiving this request, the server generates its own challenge value and returns it together with verification information bound to the shared key established during system initialization. The client verifies the authenticity of the server’s response and then sends a confirmation message that allows the server to verify the client’s legitimacy. Only after both parties have successfully validated each other’s identities will the encrypted facial matching process be executed. Although mutual authentication protocols have been extensively studied and widely deployed in practical secure communication systems, the specific mechanism adopted in this work is tailored to our setting and aims to ensure freshness, prevent replay, and resist impersonation attacks. Therefore, the above description highlights only the parts relevant to our threat model rather than reiterating well-established authentication techniques. Each authentication round uses a unique session identifier and challenge value, ensuring that previously intercepted messages cannot be reused, thereby effectively preventing replay and impersonation attacks.
- *Privacy of facial features*: This article uses the symmetric encryption algorithm SHE to encrypt facial features, ensuring that the data is protected during transmission from tampering or leakage, thereby maintaining the integrity and privacy of the data. In the identity authentication stage, the server performs similarity calculation of facial features in encrypted state to ensure that the facial features and similarity have not been tampered with or leaked, thereby ensuring the integrity of the data during processing.
- *User anonymous*: It refers to the situation where the client encrypts and uploads the user’s facial features, and the server cannot steal the plaintext information of the user’s facial features. In the identity authentication stage, the homomorphic encryption algorithm is used to calculate the similarity during facial feature matching in ciphertext state, and the user’s facial features will not be leaked.
- *Man-in-the-middle Attack*: “Man in the middle attack” is a network security threat that refers to attackers stealing sensitive information or manipulating communication content by intercepting and tampering with data streams during the communication process. In man-in-the-middle attacks, attackers typically establish fake connections between the two ends of communication, causing both parties to mistakenly believe that they are communicating directly, when in fact all communication is under the control and monitoring of the attacker.
- *Replay attack*: This article uses the SHE algorithm to generate keys and select random and prime numbers during the initialization phase, ensuring the security and randomness of the keys and providing a stable foundation for subsequent steps.
- *Insider attack*: An “insider attack” refers to the situation where employees, partners, or other authorized users within an organization use their legitimate permissions or access rights to engage in malicious behavior against the organization’s information systems, data, or resources. These behaviors may be intentional or unintentional, but they can pose a serious threat to the security, data confidentiality, and availability of the organization.

## 4. Privacy Protection Method of Face Recognition Based on Symmetric Homomorphic Encryption

This section introduces a privacy-preserving face recognition method based on symmetric homomorphic encryption, implemented on an untrusted server. The proposed solution integrates facial feature extraction, classification based on FFCM, and encrypted similarity calculation to achieve secure and efficient authentication. Within this framework, the client performs feature extraction and encryption operations, while the server stores the encrypted templates and conducts ciphertext matching. The entire process is divided into three stages—initialization, registration, and authentication—which will be described in detail in the following subsections.

### 4.1. Initialization Stage

The main task at this stage is to construct the facial feature classification structure and initialize the FFCM model required by the proposed scheme.

The server creates a face classification model based on the convolutional neural network model, and constructs an N-ary tree for storing face features based on the face classification model.

To avoid ambiguity, we explicitly state that the four CNN attribute classifiers used in FFCM are all lightweight models (with approximately 1–2 million parameters), and they are independently trained using publicly available facial attribute datasets. Their purpose is not to perform highly accurate semantic predictions, but rather to provide rough attribute classification to reduce the ciphertext search space. Their accuracy rates (depending on the attribute, approximately 78–92%) are sufficient for tree-based partitioning, and these classifiers are only stored during system initialization. During the encryption matching process, the server does not need these classifiers, thereby reducing the storage pressure.

The server obtains a large number of face image datasets and corresponding labels, which represent the category of face features, and adjusts the size and normalization of face images to ensure that all face images have the same input size and format. The server uses the Keras library to create a convolutional neural network (CNN) model for face feature classification. The convolutional neural network model includes several convolutional layers and pooling layers, and the last layer is a fully connected layer, which takes the face image dataset as the input of the convolutional neural network model. The corresponding labels are taken as output, the convolutional neural network model is trained, the number of training rounds and the batch size are set, and finally the trained face classification model is obtained.

In this paper, if the face image is classified according to face shape, skin color, nose bridge, and eyelids, the resulting face classification model includes the face shape classification model, skin color classification model, nose bridge classification model, and eyelid classification model; that is to say, there are several categories of labels set in the face image dataset, and several face classification models can be trained eventually.

The server builds an N-ary tree based on several trained face classification models, which is used to classify the user’s face features. n represents the maximum number of branches of a node in the N-ary tree, and the categories of face features are face shape, skin color, nose bridge, and eyelids. The process of building an N-tree is as follows:

(1) Define the node structure: The data domain of each node is used to store the node value of the face features (such as face shape, skin color, nose bridge, etc.). If the node has a child node, the node’s data domain also stores a pointer to the child node.
(2) Create the root node of the N-ary tree: Define the data field of the root node as the first node value used to classify face features, such as face shape classification.
(3) Add child nodes: Based on the root node, add child nodes to it, and add child nodes to existing nodes, so each node can have 1 to N child nodes.
(4) Leaf node number: If the depth (number of layers) of the N-tree is h, H-1 categories are stored, and each leaf node stores all face features that match the path from the root node to the leaf node, and the leaf node is uniquely numbered according to the path.
(5) The trained face classification model is matched to each layer node of the N-ary tree, and each layer node corresponds to a face classification model. The coding method FFCM (Face Feature Coding Method) based on face feature classification is constructed, with *P* = FFCM(*X*), where *X* represents the extracted face features and *P* represents the number of the leaf node.

The N-ary tree constructed by the server defines each leaf node as a subset, and each subset is an empty set in the initialization stage, and the face features of each user are stored while waiting for the user registration stage. The server sends the constructed N-tree to the client, which makes it convenient for the client to classify face features based on the N-tree.

### 4.2. Registration Phase

Before performing the Principal Component Analysis (PCA) projection, all face images were aligned using five facial feature points and scaled to a resolution of 256 × 256 to reduce the influence of pose and lighting variations.

**Figure 3:**
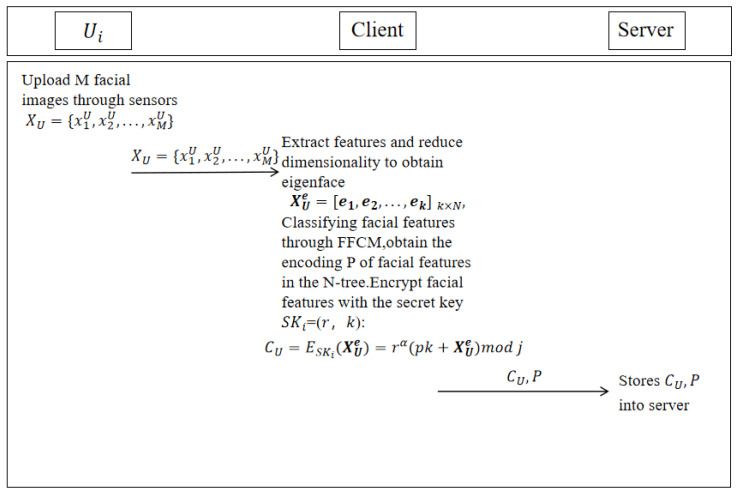
Registration phase.

As shown in Figure 3, after dimensionality reduction of the user’s face image, the client calculates the corresponding feature vector and feature value to obtain the user’s face features. After classifying and encrypting the user’s face features, these features are uploaded to the server and stored in the corresponding subsets of the N-tree.

First, the client collects M face images of user U $X_{U}=\left\{x_{1}^{U},x_{2}^{U},\ldots ,x_{M}^{U}\right\}$, adjusts the format of the face image to a 256 × 256 × 3 RGB image, and then reduces the dimensionality of the face image and extracts the face features.

Assume $X_{U}$ is a dataset of grayscale face images, and each face image $x_{i}^{U}$ is expressed as an m×n matrix, i=1,2,…,M. Transform $x_{i}^{U}$ into a one-dimensional vector $\alpha_{i}$ by each row of a connection matrix [34]:

(4) $$\;x_{i}^{U}=\left[\begin{array}{ccc} \alpha_{11} & \cdots & \alpha_{1n}\\ \vdots & \ddots & \vdots \\ \alpha_{m1} & \cdots & \alpha_{mn} \end{array}\right]\to \alpha_{i}={\left[\begin{array}{c} \alpha_{11}\\ \vdots \\ \alpha_{1n}\\ \vdots \\ \alpha_{2n}\\ \vdots \\ \alpha_{mn} \end{array}\right]}_{N\times 1}$$

where *N* represents the dimension of the face image $x_{i}^{U}$, N=m×n, $\alpha_{HI}$ represents an element in row *H*, column *I*, of an m×n matrix, where H=1,2,…,m, *I* = 1, 2, …, n; $\alpha_{i}$ is a one-dimensional vector of length N, and M such vectors $\alpha_{i}$ form the matrix $K=[\alpha_{1},\alpha_{2},\ldots ,\alpha_{M}]$.

Compute the mean vector $\varphi =\frac{1}{M}\sum_{i=1}^{M}\alpha_{i}$ of the matrix K, subtract the mean vector φ from the one-dimensional vector $\alpha_{i}$ of each face image, and obtain the central matrix $K^{\prime }=\left\{\kappa_{1},\kappa_{2},\ldots ,\kappa_{M}\right\}$, where $\kappa_{i}=\alpha_{i}-\varphi$. Then, calculate the covariance matrix C:

(5) $$C=\frac{1}{M-1}K_{i}^{\prime }K_{i}^{\prime T}$$

The size of the covariance matrix C is N×N, and *T* is the transpose of the matrix. Then, the eigenvalue decomposition of covariance matrix C is carried out; we obtain the eigenvectors $\eta_{i}$ and eigenvalues $\mu_{i}$, satisfying $C\eta_{i}=\mu_{i}\eta_{i}$.

In order to reduce the calculation cost, this paper calculates the matrix $C\prime =K^{\prime T}K^{\prime }$, where the size of the matrix C′ is defined as M×M. Generally speaking, *M* is much less than *N*; then, the eigenvector of the covariance matrix *C* can be obtained by calculating the eigenvector of the matrix C′.

In particular, if $e_{i}$ and $\lambda_{i}$ are eigenvectors and eigenvalues of the matrix C′, respectively, then we have the following:

(6) $$\begin{array}{cc} C\prime \cdot e_{i} & =\lambda_{i}\cdot e_{i}\\ K^{\prime T}\cdot K^{\prime }\cdot e_{i} & =\lambda_{i}\cdot e_{i}\\ K^{\prime }\cdot K^{\prime T}\cdot K^{\prime }\cdot e_{i} & =K^{\prime }\cdot \lambda_{i}\cdot e_{i}\\ C\cdot \left(K^{\prime }\cdot e_{i}\right) & =\lambda_{i}\cdot \left(K^{\prime }\cdot e_{i}\right) \end{array}$$

Since $C=K^{\prime }K^{\prime T}$, the eigenvector $e_{i}$ of the covariance matrix C can be obtained by calculating $k^{\prime }\cdot e_{i}$, that is, $\eta_{i}=k^{\prime }\cdot e_{i}$, eigenvalues $\mu_{i}=\lambda_{i}$. The eigenvectors of covariance matrix C are sorted from large to small, and the first *k* eigenvectors are selected to create the eigenvector matrix $X_{U}^{e}={\left[e_{1},e_{2},\ldots ,e_{k}\right]}_{k\times N}$, and the feature vector matrix $X_{U}^{e}$ is the extracted face feature of user U. Since the dimensionality-reduced data obtained through PCA will be further used for image classification, the number of retained dimensions *k* should be chosen to preserve as many principal components as possible, ensuring that the cumulative variance contribution rate approaches 95%. Then, the client inputs the face feature $X_{U}^{e}$ into the N-ary tree constructed by the server and obtains the code *P* = FFCM($X_{U}^{e}$) of face feature $X_{U}^{e}$ in the N-ary tree using the FFCM coding method.

Then, the client chooses security parameters *j*, *k*, *p*, and r∈Z that satisfy j≫k and p+k<j, finally obtaining the key SK=(r,k) and common parameter J=(j,k,p,r).

Using the key SK and a parameter α, we encrypt the face features $X_{U}^{e}$ as follows: $Enc\left(SK,X_{U}^{e}\right)\to E\left(X_{U}^{e}\right)$. Here, $E(X_{U}^{e})$ indicates the encrypted face feature ciphertext, and we have the following:

(7) $$E\left(X_{U}^{e}\right)=E\left(SK,X_{U}^{e},\alpha \right)=r^{\alpha }\left(pk+X_{U}^{e}\right)\;mod\;j$$

**Figure 4:**
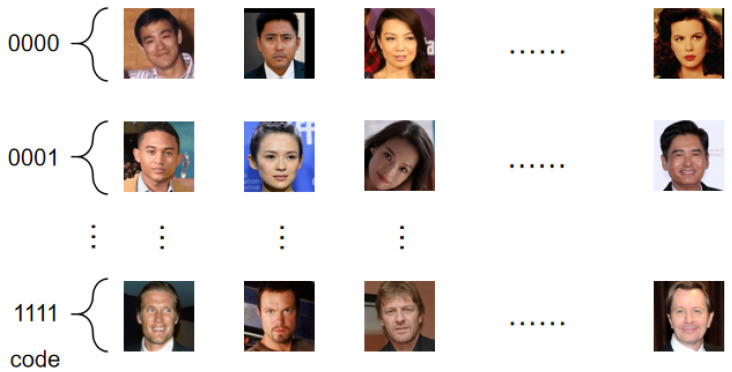
Each encoding corresponds to a subset that stores multiple encrypted facial features.

The face feature ciphertext and code $\left(X_{U}^{e},P\right)$ are sent to the server, and the server stores the face feature ciphertext in the corresponding subset according to the code *P*. As shown in Figure 4, multiple face feature ciphertexts can be stored in the subset corresponding to each encoding.

### 4.3. Authentication Phase

**Figure 5:**
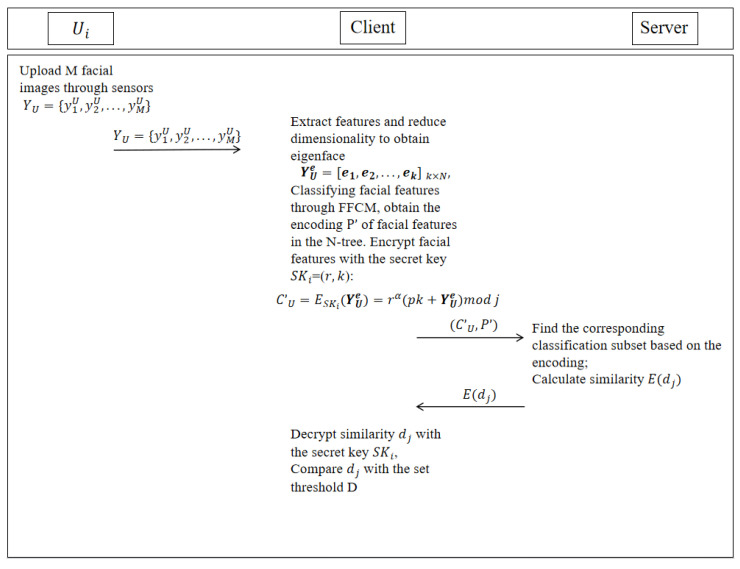
Authentication phase.

The client extracts the face features of the user to be authenticated, encrypts them, and uploads them to the server. As shown in Figure 5, the server calculates the similarity between the ciphertext of the face features of the user to be authenticated and the ciphertext stored in the N-tree, encrypts the similarity calculation result, and returns it to the client for judgment. The client determines whether the user to be authenticated has been registered.

In the identity authentication stage, the user U to be authenticated inputs the face image $Y_{U}=\left\{y_{1}^{U},y_{2}^{U},\ldots ,y_{M}^{U}\right\}$ to the client. The method of extracting the face features of the user to be authenticated and encrypting them to upload to the server is the same as that in the user registration stage; the dimension of face image $y_{M}^{U}$ is reduced through the connection matrix and transformed to a one-dimensional vector $\beta_{i}$:

(8) $$\;y_{i}^{U}=\left[\begin{array}{ccc} \beta_{11} & \cdots & \beta_{1n}\\ \vdots & \ddots & \vdots \\ \beta_{m1} & \cdots & \beta_{mn} \end{array}\right]\to \beta_{i}={\left[\begin{array}{c} \beta_{11}\\ \vdots \\ \beta_{1n}\\ \vdots \\ \beta_{2n}\\ \vdots \\ \beta_{mn} \end{array}\right]}_{N\times 1}$$

Subtract the mean vector $\delta^{\prime }$ from the one-dimensional vector $\beta_{i}$ of each face image, and obtain the central matrix $\Gamma =\left\{\Gamma_{1},\Gamma_{2},\ldots ,\Gamma_{M}\right\}$, where $\Gamma_{i}=\beta_{i}-\delta^{\prime }\left(i=1,2,\ldots ,M\right)$. Then, calculate the covariance matrix C:

(9) $$C=\frac{1}{M-1}\Gamma_{i}\Gamma_{i}^{T}$$

The eigenvector $\eta_{i}$ and eigenvalue $\mu_{i}$ are obtained, as well as the face feature $Y_{U}^{e}$ of user U to be authenticated.

The client classifies face feature $Y_{U}^{e}$ through an N-ary tree to obtain code $P^{\prime }$ and encrypts face feature $Y_{U}^{e}$ through the symmetric encryption algorithm with the SK=(r,k) of *U*:

(10) $$E\left(Y_{U}^{e}\right)=E\left(SK,Y_{U}^{e},\alpha \right)=r^{\alpha }\left(pk+Y_{U}^{e}\right)\;mod\;j$$

Then, it uploads face feature ciphertext and code $\left(E\left(Y_{U}^{e}\right),P^{\prime }\right)$ to the server. The server traverses all stored face feature ciphertext $E(X_{i}^{e})$ in the corresponding subset according to code $P^{\prime }$, where $E(X_{i}^{e})$ represents the $i^{th}$ personal face feature ciphertext stored in a subset, which stores S personal face feature ciphertexts, i=1,2,…,S. Calculate the similarity between each face feature ciphertext $E(X_{i}^{e})$ in the subset and the face feature ciphertext $E(Y_{i}^{e})$ of the user to be authenticated:

(11) $$\begin{array}{cc} E(d_{i}) & =E\left(1\right)+E\left(-1\right)\cdot \frac{E(X_{i}^{e})}{||E\left(X_{i}^{e}\right)\left|\right|}\cdot \frac{E(Y_{U}^{e})}{||E\left(Y_{U}^{e}\right)\left|\right|}\\ & =E(1-\frac{X_{i}^{e}\cdot Y_{U}^{e}}{∥X_{i}^{e}∥\cdot ∥Y_{U}^{e}∥}) \end{array}$$

where $E(d_{i})$ represents the similarity between the $i_{th}$ personal face feature ciphertext stored in the subset and the face feature ciphertext of the authenticated user, E(1) means to encrypt 1 in the cosine similarity, and E(−1) means to encrypt −1 in the cosine similarity.

The server sends the set of similarity calculation results $E(d_{i})$ to the client, i=1,2,…,S. Since $E(d_{i})$ is ciphertext, the client uses the key SK to decrypt $E(d_{i})$, thus obtaining the plaintext similarity result $d_{i}$:

(12) $$\begin{array}{l} d_{i}^{\prime }=\left(E\left(d_{i}\right)r^{-\alpha }\;mod\;j\right)\;mod\;k\\ d_{i}=Dec\left(E\left(d_{i}\right)\right)=\left\{\begin{array}{cc} & d_{i}^{\prime },d_{i}^{\prime }<\frac{k}{2}\\ d_{i}^{\prime }- & k,other \end{array}\right. \end{array}$$

Compare $d_{j}$ with the threshold D. If $d_{j}⩽D$, the user to be authenticated is authenticated. Otherwise, the client sends a message to the server indicating that the authentication fails.

The server first calculates the similarity in other subsets of this subset contract layer and then sends it to the client for authentication. If the authentication still fails, the server calculates the similarity in the layer where the parent node of this subset resides. After traversing all nodes, the authentication still fails, and the user authentication fails.

## 5. Security Analysis

### 5.1. Adversarial Model

We consider an adversary *A* that controls the radio communication channel between the client CT and the server *S*. *A* is required to model the following set of queries within probabilistic polynomial time (PPT).

(1) *Send* (S, $m_{1}$, $n_{1}$): This query simulates the adversary’s ability to act like a legitimate tag. In this context, *A* sends $m_{1}$ and receives $n_{1}$ from *S*.
(2) *Query* (CT, $n_{2}$, $m_{2}$): This query simulates the adversary’s ability to interrogate the client. To achieve this, *A* sends $n_{2}$ to CT and receives $m_{2}$ in response.
(3) *Execute* (CT, S): This query simulates the adversary’s ability to continuously observe the radio channel between CT and *S*. In this scenario, *A* must intercept the channel during the execution of the protocol instance between CT and *S*.
(4) *Reveal* (T): This query models *A*’s ability to access the contents of the server’s memory. In other words, this query simulates the adversary’s capability to compromise the server and retrieve the secrets stored in its memory.

It is important to note that *A* can invoke the Send, Query, and Execute queries an arbitrary polynomial number of times but can only call the Reveal query once. Furthermore, based on the previously discussed definition of the adversary and the formal adversarial model outlined above, Type 1 adversaries can invoke all oracle queries except for the Reveal oracle. In contrast, Type 2 adversaries have the capability to invoke all oracle queries, including the Reveal oracle.

**Theorem** **1.** *(Untraceability): In the proposed authentication protocol, the client is universally untraceable.*

**Proof.**  In our face authentication system, the client is untraceable if adversary *A* cannot associate its two successful authentication requests with the valid server *S*. This can be modeled as the following game between challenger *C* and adversary *A* within our face authentication system. We assume that the capabilities of both *C* and *A* do not exceed those of polynomial-time algorithms, as follows:

(1) *C* selects a valid server *S* and two clients $CT_{1}$ and $CT_{2}$.
(2) *A* invokes the oracle queries—*Send*, *Query*, and *Execute*—on *S* as well as on $CT_{1}$ and $CT_{2}$, for an arbitrary polynomial number of times.
(3) After invoking the oracles, *A* notifies *C*.
(4) *C* randomly selects one of the clients, $CT_{1}$ or $CT_{2}$.
(5) *A* invokes the following oracle queries on *S* and the selected CT: *Send*, *Query*, and *Execute*.
(6) *A* makes a prediction T′; if T′=T, then *A* wins the game.

 □

In this case, the advantage of a successful guess is defined as $Adv_{A}$ = 2 × (*Pr*[T’ = T] −$\frac{1}{2}$). If the adversary *A* has no advantage over random guessing and $\mathrm{Pr}\left[T\prime =T\right]=\frac{1}{2}$, then *Adv*_*A*_=0, indicating that the client is deemed untraceable.

We will follow the aforementioned framework to ensure the universal untraceability of clients in the proposed authentication protocol. In this context, we assume that client C successfully executes the authentication process between each client $CT_{1}$ and $CT_{2}$ and the server *S*. Then, *C* randomly selects a client $CT_{i}$ (i = 1, 2) and hands it over to *A*. *A* makes her prediction T′ after invoking the following oracles: *Send*, *Query* and *Execute*. Since *A* is unable to generate the correct identity ID, *P*, she has no choice but to make a random guess $\mathrm{Pr}\left[T\prime =T\right]-\frac{1}{2}$, and according to the equation for *Adv_A_* above, the adversary’s advantage is zero. Thus, our proposed lightweight face authentication protocol guarantees universal untraceability.

**Theorem** **2.** *The proposed protocol achieves mutual authentication.*

**Proof.**  Adversary *A* may attempt to authenticate herself as a legitimate client, which can be modeled through the following game between *A* and challenger *C*.

(1) *C* selects a valid server *S* and client CT.
(2) *A* invokes the oracle queries—*Send*, *Query*, and *Execute*—on *S* and CT, for an arbitrary polynomial number of times.
(3) After invoking the oracles, *A* notifies *C*.
(4) *A* invokes the Send oracle to simulate the client.
(5) If *A* is able to prove herself as a legitimate client, then *A* wins the game.

 □

Now, to prove her legitimacy, *A* must respond to the server’s queries. To accomplish this, *A* needs to send a valid face feature encoding and also generate a valid face feature ciphertext. However, since *A* is not a legitimate client, she does not possess the correct face feature encoding method FFCM from the server. As a result, she cannot generate a valid encoding, which means she cannot impersonate a legitimate client.

(*Security*) Assuming the server honestly executes the operations, it generates the required face feature encoding method for the system. In our scheme, for a non-uniform polynomial-time adversary *A* or untrusted user, if the face feature data is not registered on the server, it is computationally infeasible to forge a valid credential that can be verified by the client.

**Proof.**  The security proof of the scheme implies that if the scheme is compromised, it would lead to a solution for the integer factorization problem. Thus, this implies that the probability of an attacker successfully authenticating under limited computational resources is negligible. To prove the security, it suffices to demonstrate the following Lemma 1. □

**Lemma** **1.** *If VerifyEval is a random oracle and there exists an adversary A that can decrypt the face feature ciphertext with a non-negligible probability in PPT, then A must possess a knowledge extractor capable of solving the integer factorization problem.*

**Proof.**  Assume there exists such an adversary *A*. Given an instance of the integer factorization problem to adversary *B*, N=p×q, *B* is also granted oracle access to the face feature encoding method FFCM for randomly registered users. *B* sends a randomly selected challenge *e* to *A*. *A* is expected to ultimately output the response *P* for the challenge. □

*B* initializes a set BSET, which includes a randomly selected face feature image $X_{u}$, obtains the class label $P_{B}$, and adds ($X_{U_{B}}^{e}$, $P_{B}$) to BSET. $X_{U_{B}}^{e}$ is the feature vector obtained from the dimensionality reduction of the face feature image $X_{u}$. When *A* queries each classification function at value $\omega_{A}$, *B* performs the following actions:

If the tuple ($X_{U_{A}}^{e}$,$P_{A}$) already exists in the corresponding tuple set, respond with $P_{A}$. Otherwise, it queries $P\prime_{A}\leftarrow \mathrm{FFCM}$($X_{U_{A}}^{e}$), adds ($X_{U_{A}}^{e}$, $P\prime_{A}$) to BSET, and replies to *A* with $P\prime_{A}$. If $P_{A}=P_{B}$, then *B* outputs a failure. Otherwise, *B* receives $P_{A}$ and sends it to *A*.

(*Privacy protection*) Apart from the user’s face feature ciphertext and encoding, the server cannot obtain any useful sensitive information from the user or the client.

**Proof.**  The privacy protection proof of the scheme demonstrates that the records between the server, user devices, and the registration and authentication protocol of the scheme do not leak any knowledge regarding the face feature information, for the following reasons:

(1) In this scheme, the client strictly adheres to the computational principles of symmetric homomorphic encryption algorithms. Even if the server is honest yet curious, it cannot decrypt the face feature ciphertext without knowledge of the client’s key.
(2) In the registration and authentication protocol of our scheme, there is no interaction between the user and the server; both only interact with the client. Therefore, the server cannot obtain any face feature information from the user.

 □

To further clarify the security measures of the proposed solution, the following analysis is provided. The security of this solution is evaluated under the assumption that the server is “honest but curious”. Since all facial features and similarity values have been encrypted before transmission, the server cannot obtain any plaintext biometric information. Moreover, the two-way authentication mechanism and session-based protection measures effectively prevent replay and impersonation attacks. Even in the case of internal threats, the encrypted database cannot be meaningfully associated to re-identify the user. Therefore, this solution ensures confidentiality and strong privacy protection throughout the authentication process.

### 5.2. Privacy Implications of FFCM Encoding

Although the Facial Feature Coding Method (FFCM) improves matching efficiency by classifying facial features based on attributes such as face shape and skin color, if this process directly exposes such semantic information to attackers, it may bring additional privacy risks. Specifically, attackers observing the classification results may attempt to infer rough personal characteristics or conduct portrait-based analyses.

To reduce this potential risk of privacy leakage, the proposed solution avoids storing explicit facial feature information on the server. Instead, it only uses the abstract classification indices generated by the FFCM process to locate the encrypted subsets, and the correspondence between these indices and the actual facial features is only retained on the client side. Moreover, all subsequent matching operations are performed on encrypted data, further preventing attackers from obtaining meaningful personal information from the FFCM encoding.

## 6. Experiment

In this section, we first introduce the datasets used in the experiments and analyze the recognition performance of the proposed scheme. We then evaluate the impact of different parameters on face recognition performance and analyze the computational and communication costs of the scheme. Finally, we compare the face recognition performance of our scheme with that of other methods.

In this work, the entropy calculation refers to the overall cost required to complete an authentication process, which includes the time for feature extraction, encryption, ciphertext matching, and similarity calculation. Specifically, the cost is quantified by the total number of arithmetic operations and execution time consumed in these steps.

### 6.1. Dataset

We used three grayscale graph datasets, ORL, CASIA and CelebA-HQ. The ORL dataset consisted of 40 people, each of whom had 10 photos of different shapes and at different times. The dataset CASIA contains 500 people with 5 images each, for a total of 2500 images. The CelebA-HQ dataset consisted of 200 individuals, each of whom had 10 images taken in different poses, expressions, and lighting conditions. The relevant details of each dataset are shown in Table 3.

Although the ORL, CASIA, and CelebA-HQ datasets enable us to evaluate the basic effectiveness of the proposed privacy-preserving identification scheme, their relatively small scale limits the statistical significance of the results. Due to the limitations of computing resources and the need to train multiple attribute classifiers and encryption domain components, experiments cannot be conducted on large-scale benchmark tests (such as AgeDB, LFW, or the IJB series datasets) at present. In future work, we plan to extend the evaluation to larger and more diverse datasets to further verify the robustness and scalability of the proposed method.

### 6.2. Recognition Performance

The scheme achieves remarkable performance on all the above datasets. The experimental results are shown in Table 4. In the experiment on each dataset, 20 face images of the same type as the dataset are selected to build the feature surface, with CEV = 96% (0⩽CEV⩽1, where Cumulative Explained Variance (CEV) indicates variance information explained by the principal components). A total of 20% of this dataset, along with 4 to 10 unknown images, was selected for identification testing for registered and unregistered users, respectively. The thresholds *T* for the ORL, CASIA, and CelebA-HQ datasets are 0.60, 0.40, and 0.60, respectively.

For the ORL, CASIA, and CelebA-HQ datasets, the recognition accuracy is above 97%. In addition, to verify the robustness of the scheme, we compiled the CASIA and CelebA-HQ datasets into the large dataset CCHQ. In more detail, 30% of the images from the CASIA and CelebA-HQ dataset were included in the image collection, producing 750 users with a total of 5000 images. The threshold in the dataset CCHQ is 0.10. The overall recognition accuracy reached 98.325%.

**Figure 6:**
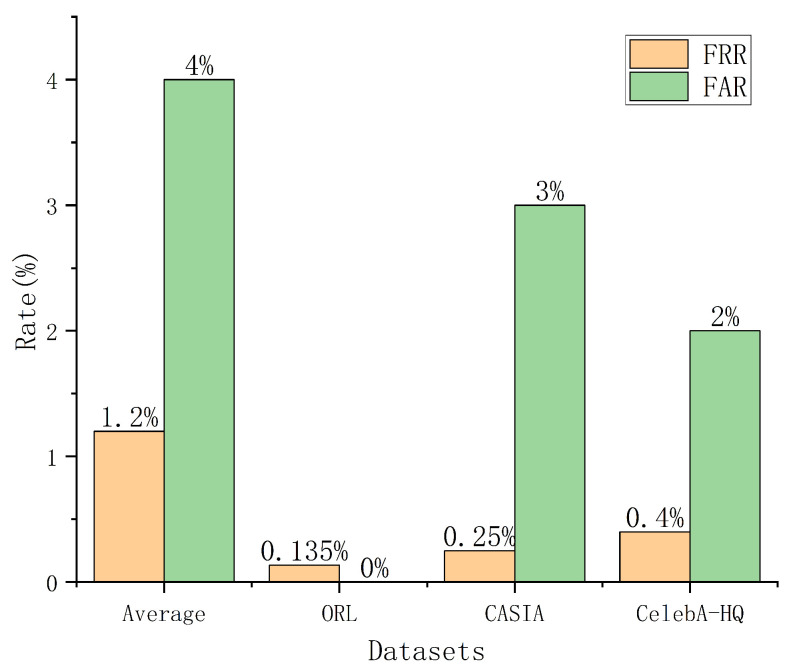
FRR and FAR comparison across datasets.

Our scheme demonstrates excellent performance in terms of False Acceptance Rate (FAR) and False Rejection Rate (FRR). For example, the ORL dataset’s test set consists of 74 registered users and 6 unregistered users, the CASIA dataset’s test set consists of 2000 registered users and 100 unregistered users, and the CelebA-HQ dataset’s test set includes 1500 registered users and 50 unregistered users. In these datasets, only one registered user was incorrectly rejected in the ORL dataset, five registered users were incorrectly rejected in the CASIA dataset, and six registered users were falsely rejected in the CelebA-HQ dataset, resulting in FRRs of 0.135%, 0.25%, and 0.4%, respectively, as shown in Figure 6. Additionally, all unregistered users in the ORL dataset were successfully rejected, leading to an FAR of 0%. In contrast, three unregistered users in the CASIA dataset were mistakenly accepted and one unregistered user in the CelebA-HQ dataset was mistakenly accepted, yielding an FAR of 3% and 2%.

### 6.3. Correlation Parameter Analysis

#### 6.3.1. Feature Face Calculation Parameters

The impact of different parameters related to the feature face provided by the server on face recognition performance was discussed in both scenarios.

**Figure 7:**
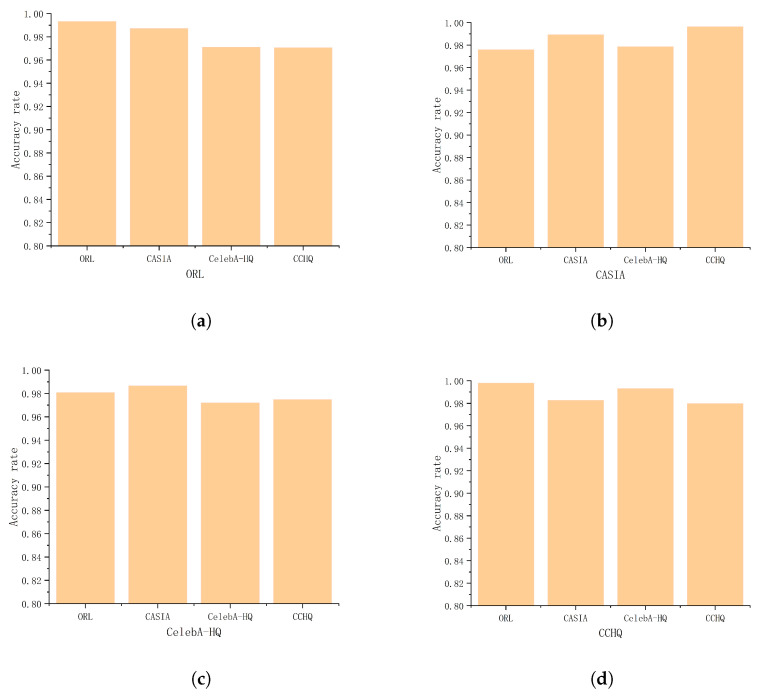
Determining the impact of feature face image types on face recognition performance. (**a**) ORL, (**b**) CASIA, (**c**) CelebA-HQ, and (**d**) CCHQ.

The first scenario explores the impact of the server using different types of datasets to construct feature faces on face recognition performance. We used the ORL, CASIA, CelebA-HQ, and CCHQ datasets to investigate the impact of constructing feature faces from different types of images on recognition accuracy. For instance, in the ORL dataset, we selected 20 images (though these images have no bearing on data collection and image recognition, they can be regarded as images of the same type), and, respectively, utilized 20 images from the CASIA, CelebA-HQ, and CCHQ datasets to create eigenfaces. We set CEV = 96%. Select 20% of registered and unregistered users from the dataset, and select 4–10 unknown images for face recognition testing for each group. The face recognition accuracy for each dataset using feature faces established from other datasets is shown in Figure 7.

As seen in Figure 7, for the ORL, CASIA, CelebA-HQ, and CCHQ datasets, the recognition accuracy for all datasets exceeds 97% when using different datasets to construct feature faces. Therefore, using different types of images to construct feature faces has a minimal impact on recognition performance.

**Figure 8:**
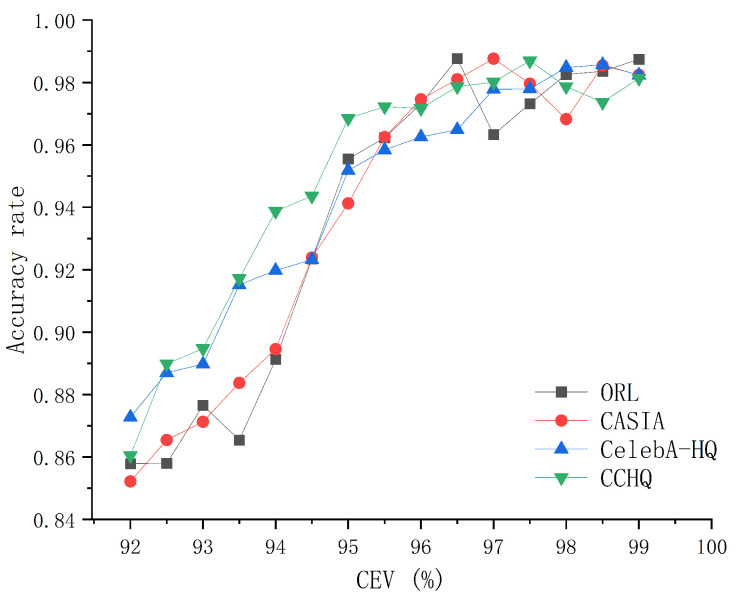
The impact of the CEV value on face recognition performance.

The second scenario involves the effect of the CEV value (the proportion of retained information) on the recognition accuracy after dimensionality reduction. We will discuss this using the ORL, CASIA, CelebA-HQ, and CCHQ datasets as examples. In each dataset, we select 20 face images that are of the same type as the dataset to construct the feature face. The impact of different CEV values on face recognition performance across various datasets is illustrated in Figure 8.

**Figure 9:**
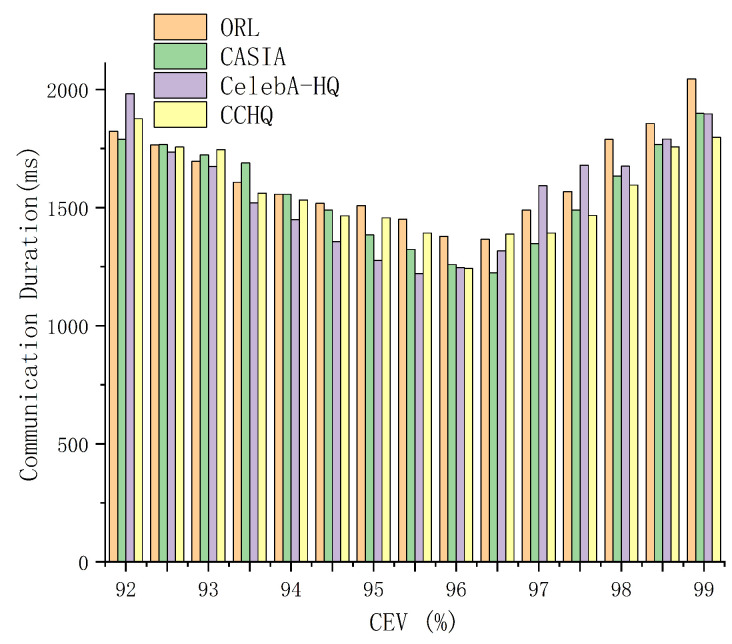
The impact of different CEV values on communication duration.

The data in Figure 8 indicate that for the ORL, CASIA, CelebA-HQ, and CCHQ datasets, as the CEV value increases, the recognition accuracy also improves. When the CEV value reaches or exceeds 96.5%, the recognition accuracy remains at a high level and stabilizes. However, during the experiments, we observed that when the CEV value exceeds a certain threshold, the communication time increases. The impact of different CEV values on communication duration is shown in Figure 9. As observed from Figure 9, the communication time is minimized when the CEV value is approximately 96. Therefore, the optimal CEV value we select is 96%.

#### 6.3.2. The Number of Classification Models

**Figure 10:**
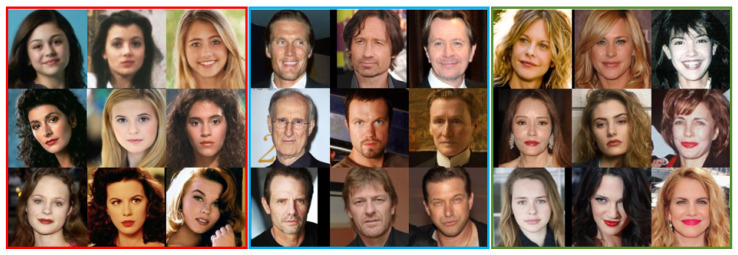
Result images using the CASIA dataset, with each figure employing one of six classification models.

**Figure 11:**
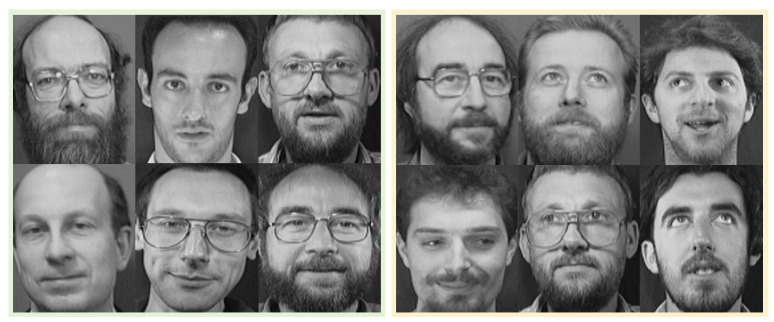
Result images using the ORL dataset, with each figure employing one of three classification models.

**Figure 12:**
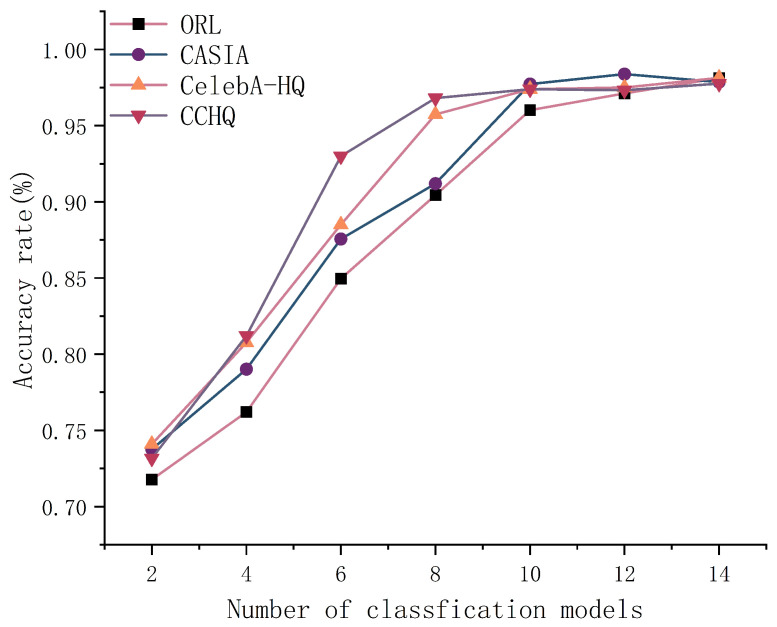
The effect of model number on accuracy.

We used the ORL, CASIA, CelebA-HQ, and CCHQ datasets to verify the impact of the number of classification models used across different datasets on recognition accuracy. For each dataset, we selected 20 face images of the same type to construct the feature face, with CEV = 96%. Figure 10 displays partial result images using the CASIA dataset, with each figure employing one of six classification models. Specifically, the classification model indicated by the red box on the left in Figure 10 is characterized by “oval face shape, long hair, light eyebrows, moderate cheekbones, high nose bridge, and thick lips.” The model in the blue box in the middle corresponds to features such as “square face shape, short hair, light eyebrows, moderate cheekbones, high nose bridge, and thin lips,” while the model in the green box on the right is defined by “round face shape, long hair, thick eyebrows, moderate cheekbones, high nose bridge, and thick lips.” Figure 11 shows partial result images from the ORL dataset, with each image representing one of the three classification models. The left part corresponds to a model characterized by “high hairline, short hair, and narrow eye spacing,” and the right part corresponds to a model with features including “facial beard, short hair, and high nose bridge.” Figure 12 illustrates the face recognition performance across datasets when the number of classification models is set from 2 to 14.

**Figure 13:**
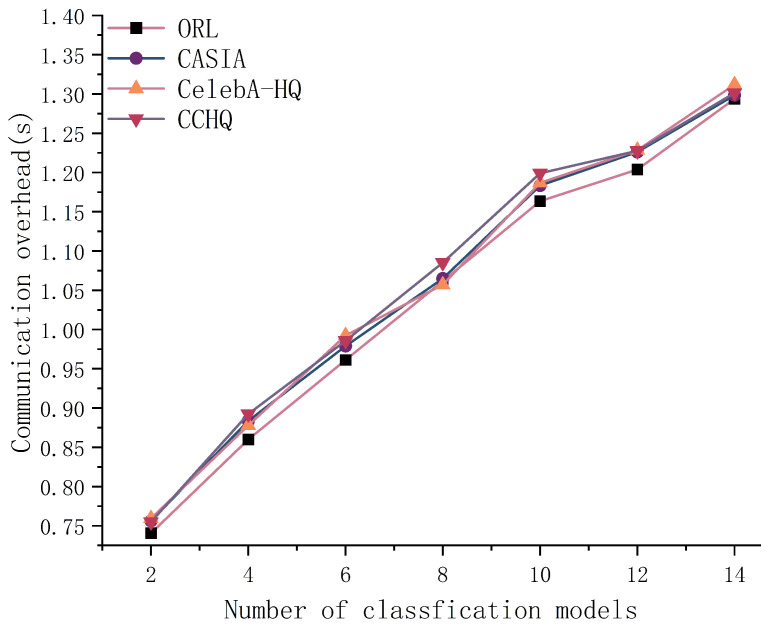
The effect of the number of classification models on communication overhead.

As shown in Figure 12, the face recognition performance peaks and remains stable when the number of classification models is around 10. However, as the number of classification models increases, the communication overhead also rises when facial image features are classified using FFCM. As demonstrated in Figure 13, when the number of classification models exceeds 12, the average communication overhead across the four datasets reaches 1.3 s. Therefore, a higher number of classification models does not necessarily lead to better performance.

### 6.4. Cost Analysis

This section will compare the computational efficiency of our proposed scheme with several related schemes [12,16]. A key reason for this choice is that these datasets employ the same similarity comparison techniques as our proposed face authentication protocol. This enables us to make meaningful and useful comparisons regarding performance. In contrast, other protocols that use different methods for similarity comparison may have varying performance parameters, making meaningful comparisons difficult. Our platform includes a mobile device and a personal computer. The mobile device is an iPhone 14 Pro, equipped with a five-core A16 processor and 6 GB of RAM, and running iOS 17.7. The personal computer is an ASUS with an i5-12400F 2.50 GHz processor, 16 GB of RAM, and an NVIDIA 4060 graphics card, running Windows 11. Table 5 displays the symbols used to measure computational costs and their corresponding execution times.

(1) $T_{ext}$: The time taken to extract features and reduce them to feature vectors;
(2) $T_{cff}$: The time taken to classify face features;
(3) $T_{Enc}$: The time taken to encrypt face features;
(4) $T_{sc}$: The time taken to retrieve face features and perform similarity comparisons;
(5) $T_{Dec}$: The time taken to decrypt face features.

Table 5 and Table 6 focus on the computational efficiency during the authentication phase. It can be seen that, at the client side, the computational cost of the proposed scheme is lower than that of the previously related schemes [12,16]. On the server side, due to the use of the FFCM classification method, the matching search time is significantly reduced, resulting in a computational cost for our scheme that is comparable to that of the previously related schemes. Additionally, since we use a symmetric encryption algorithm, our scheme is generally faster than theirs overall.

The face recognition process is typically real-time, making the efficiency of the recognition scheme crucial and necessitating effective assurance. Inspired by the works of Cozza et al. [37] and Guarino et al. [38], we analyze the performance of this scheme in terms of computational and communication costs. The results indicate that the proposed scheme can be implemented efficiently with sufficiently low computational costs. For instance, disregarding network transmission delays, the average computational time for processing a recognition request is 1.22 s, comprising two parts: 0.624 s on the client side and 0.596 s on the server side.

### 6.5. Comparison with Other Schemes

In this section, we evaluate the performance of different face recognition schemes using the ORL dataset. First, we consider the privacy-preserving face recognition method (PPFR) based on encrypted protocols proposed by Sadeghi et al. [25]. While PPFR provides strong privacy guarantees, it achieves an acceptable recognition accuracy of 96.4%. In comparison, our scheme achieves an accuracy of 98.582%, which is 2% higher than that of PPFR, demonstrating superior performance.

Additionally, we compare the performance of the revocable reusable scheme (PRIFACE) proposed by Lei et al. [16] and the eigenfaces algorithm, which utilizes only feature faces. The former is an improvement based on random masking techniques. The comparison results of our scheme with PRIFACE and Eigenfaces are shown in Table 7. The results indicate that our scheme achieves higher and more lightweight face recognition performance compared to the other solutions.

### 6.6. Comparison with Mainstream Homomorphic Encryption Schemes

To further evaluate the practicality of the proposed symmetric homomorphic encryption (SHE) scheme, we compared it through discussion with several representative generic homomorphic encryption schemes commonly used in privacy-preserving computing. These schemes include BFV [39], BGV [40], and CKKS [41]. These schemes have been widely applied in libraries such as Microsoft SEAL and TenSEAL, and are considered leading in the field of encrypted domain machine learning.

Although BFV [39], BGV [40], and CKKS [41] have stronger algebraic expression capabilities and noise management capabilities, they usually require operations in large polynomial rings, resulting in significantly higher computational and memory costs. In contrast, the SHE scheme used in this paper relies on modular integer operations, making encryption and ciphertext operations more lightweight and more suitable for resource-constrained facial authentication scenarios. To clearly illustrate these distinctions, Table 8 summarizes the conceptual differences between the proposed SHE scheme and the mainstream BFV [39], BGV [40], and CKKS [41] schemes. While the mainstream schemes offer robust polynomial arithmetic, their high complexity renders them less suitable for lightweight face recognition tasks compared to our SHE scheme, which utilizes efficient integer modulus operations.

Since our authentication system uses cosine similarity based on the facial features after dimensionality reduction through Principal Component Analysis (PCA), precise integer domain calculation is sufficient, while the homomorphic scheme based on polynomials would incur unnecessary overhead. Furthermore, the matching times reported by existing face recognition studies based on HE technology (such as CipherFace [42]) range from several hundred milliseconds to several seconds. However, our method based on SHE has significantly lower computation time due to its simpler algorithm. This comparison indicates that the proposed SHE scheme provides a better balance between computational efficiency and privacy protection for lightweight real-authentication scenarios.

## 7. Conclusions and Future Work

This paper designs a lightweight privacy-preserving face recognition scheme based on symmetric homomorphic encryption, which addresses the increasing entropy in face recognition systems while ensuring high performance in real-world scenarios. The scheme effectively prevents users’ face information from being compromised by malicious servers. We hope that this research can be applied to privacy protection in identity authentication, such as avoiding the capture of the user’s real face image by malicious servers or third parties during the face recognition process, and ensuring that decision-making authority in identity authentication resides with the client, thereby preserving the credibility of user information and face recognition.

The most innovative aspect of our scheme lies in the similarity calculation performed in the ciphertext state on the server. In other words, even if the server is malicious or semi-honest, the similarity calculation in ciphertext will not expose the user’s facial feature information. The similarity ciphertext is then sent back to the client for threshold comparison after decryption, ensuring that the decision-making power for face recognition stays with the client. Additionally, we employ the Face Feature Coding Method (FFCM) to classify user facial features, which significantly speeds up the server’s search for matches. Compared to existing solutions, our approach demonstrates superior identification performance and reduced computational entropy, offering better efficiency at a lower cost.

Although the proposed solution demonstrated excellent results in terms of privacy protection and authentication efficiency, due to time constraints and limitations of available computing resources, the current experimental evaluation was based solely on a relatively limited dataset. Therefore, in this study, it was not possible to fully explore the diversity of the user population and large-scale deployment scenarios.

In future work, we plan to expand the evaluation scope to larger-scale and more diverse facial datasets, including scenarios with different ages, different lighting conditions, and different postures, to further verify the scalability and robustness of the proposed solution. Additionally, more comprehensive experiments will be conducted, involving a wider range of user populations and real application environments, to conduct a more comprehensive evaluation of the system’s performance and practicality. One important direction for future research is to integrate deep face encoders with homomorphic-friendly properties (such as variants of ArcFace-lite) to enhance the recognition stability on heterogeneous datasets.

## Figures and Tables

**Table 1:** The key features, advantages, and limitations of different privacy-preserving face recognition approaches.

Approach Category	Key Features	Advantages	Limitations
Anonymization Methods [9,17,18,19,20,21,22,23]	Employing conventional techniques such as blurring, pixelation, and noise addition, or utilizing learnable models based on face swapping.	The reversible framework supports data reuse and is adaptable to multiple scenarios.	Some methods (such as the ISED architecture) require the allocation of exclusive keys for each identity, which poses key management challenges in large-scale systems.
Encryption Methods [24,25,26,27,28]	Based on mathematical algorithms (such as secure multi-party computation, SMPC), the facial data/attributes are encoded to generate ciphertext.	The privacy protection is highly effective, completely preventing the leakage of original data at the mathematical level.	The computational complexity is high, and the processes of encryption/decryption and ciphertext matching take a long time, making it difficult to adapt to real-time scenarios.
Differential Privacy Methods [29,30,31,32]	Controllable noise (such as Laplace and Gaussian noise) is added during the facial feature extraction/model training stage, and the noise intensity is controlled through a “privacy budget”.	Low computational cost, lightweight noise generation and addition process, suitable for resource-constrained devices such as mobile terminals.	There exists a “privacy–accuracy trade-off” contradiction: if ϵ is too small (strong privacy), the noise will be too large, leading to an increase in the False Rejection Rate; if ϵ is too large (high accuracy), the strength of privacy protection will be insufficient.

**Table 2:** Notations.

Notation	Descriptions
Φ	grayscale face image dataset
$\Phi_{i}$	m∗n matrix
$K^{\prime }$	the mean vector of the matrix K
C	the covariance matrix
$Y_{U}^{e}$	feature face vector
$X_{U}$	face images of user U
$E(X_{U}^{e})$	encrypted face feature ciphertext
$Y_{U}$	face images of user U to be authenticated
$E(Y_{U}^{e}$)	encrypted face feature ciphertext of user U to be authenticated
$E(d_{i})$	the similarity

**Table 3:** Face image dataset.

Name	Individuals	Total Face Images	Resolution	Image Type
ORL	40	400	92 ∗ 112	Gray image
CASIA	500	2500	256 ∗ 256	Color image
CelebA-HQ	200	2000	256 ∗ 256	Color image

**Table 4:** Face recognition performance for different datasets.

Database	Precision	Recall	Cost (ms)
ORL	0.98531	1.00	1004.246
CASIA	0.99673	1.00	1257.917
CelebA	0.97540	1.00	1219.514

**Table 5:** Operation execution time (ms).

Symbol	The Client	The Server
$T_{ext}$	235.245	-
$T_{cff}$	0.032	-
$T_{Enc}$	264.834	-
$T_{sc}$	-	596.234
$T_{Dec}$	123.983	1.00

**Table 6:** Comparison of communication overhead.

Scheme	The User	The Client	The Server	Total Cost (ms)
[16]	$T_{add}$ + $T_{mul}$	-	$T_{add}$ + $T_{mul}$ + $T_{inv}$	2281
[12]	-	$T_{ext}$+$T_{Enc}$+$T_{Dec}$	$T_{sc}$	1299.73
Ours	-	$T_{ext}$+$T_{cff}$+$T_{Enc}$+$T_{Dec}$	$T_{sc}$	1220.328

$T_{add}$: time overhead for modular addition; $T_{mul}$: time overhead for modular multiplication; $T_{inv}$: time overhead for modular inversion; $T_{pow}$: time complexity of modular exponentiation. (Note: In the proposed solution, users do not directly participate in the calculation process; all operations are carried out by the client on behalf of the users. Therefore, the cost of the user end has been included in the client operations and is not listed separately.)

**Table 7:** Face recognition performance on the ORL dataset compared to other methods.

Method	Precision	Recall	F1-Score
Eigenfaces	0.96250	1.00	0.97468
PRIFACE	0.93330	1.00	0.89788
FFCM	0.98582	1.00	0.98548

$T_{add}$: time overhead for modular addition; $T_{mul}$: time overhead for modular multiplication; $T_{inv}$: time overhead for modular inversion.

**Table 8:** Comparison of different schemes.

Scheme	Ciphertext Structure	Supported Operations	Complexity	Suitability for Face Recognition
BFV [39]	Polynomial modulus (RNS)	Exact addition/ multiplication	High	Heavyweight; high latency for vector operations
BGV [40]	Polynomial modulus with modulus switching	Exact addition/multiplication	High	Efficient but still computationally expensive for real-time authentication
CKKS [41]	Polynomial modulus with approximate arithmetic	Approximate addition/multiplication	High	Good for neural inference, but expensive for cosine similarity
Proposed SHE	Integer modulus	Exact addition/multiplication	Low	Lightweight and well-suited for 1D feature vector matching

## Data Availability

Data are contained within the article.
